# Biomagnification and potential health effects of per- and polyfluoroalkyl substances (PFAS) in a terrestrial food web

**DOI:** 10.1038/s41598-025-16395-6

**Published:** 2025-08-23

**Authors:** Frauke Ecke, Bjørnar Ytrehus, Magnus Evander, Birger Hörnfeldt, Alexandra Leijon, Jonas Malmsten, Aleksandra Skrobonja, Lutz Ahrens

**Affiliations:** 1 Organismal and Evolutionary Biology Research Programme, Faculty of Biological and Environmental Sciences, PO Box 65, FI-00014 University of Helsinki, Finland; 2 Department of Wildlife, Fish, and Environmental Studies, Swedish University of Agricultural Sciences (SLU), Umeå, 901 83 Sweden; 3 Norwegian Veterinary Institute, Ås, 1430 Norway; 4 Department of Animal Biosciences, Swedish University of Agricultural Sciences (SLU), Uppsala, 750 07 Sweden; 5 Department of Clinical Microbiology, Umeå University, Umeå, 901 85 Sweden; 6 Department of Pathology and Wildlife Diseases, Swedish Veterinary Agency, Ulls väg 2B, Uppsala, 751 89 Sweden; 7 Department of Aquatic Sciences and Assessment, Swedish University of Agricultural Sciences (SLU), Uppsala, 750 07 Sweden; 8 Department of Physical Chemistry, Vinca Institute of Nuclear Sciences, University of Belgrade, Belgrade, 11351 Serbia

**Keywords:** Bank vole, Bioaccumulation, Frösön, Histopathology, PFOS, PUUV, Ecosystem ecology, Zoology, Ecology, Infectious diseases, Environmental monitoring

## Abstract

Per- and polyfluoroalkyl substances (PFAS) are persistent organic pollutants with bioaccumulative, biomagnifying and toxic potential, and largely unknown fate and health effects in terrestrial wildlife. In a contaminated area, we studied PFAS in a terrestrial food web including soil, mushrooms, berries, and wild-living herbivores (bank vole and two ungulate species) and a specialized predator (Tengmalm’s owl). In addition, in voles, we studied potential PFAS-induced liver damage and infection with a zoonotic pathogen. Concentrations of PFAS in vole livers were among the highest reported in terrestrial wildlife. Results suggest biomagnification of PFAS from soil, mushrooms, and berries to bank voles, from mushrooms to ungulates and from voles to the owl. Most vole livers showed diffuse cytoplasmic granulation, ranging from mild to severe, as well as mild and variable hepatocellular hypertrophy. We found high prevalence of *Orthohantavirus puumalaense* infection in bank voles, highlighting the possibility of PFAS-induced infection susceptibility. Our study supports that terrestrial wildlife magnifies PFAS from the environment and highlights largely unexplored yet worrying effects on wildlife health.

**Supplementary Information:**

The online version contains supplementary material available at 10.1038/s41598-025-16395-6.

## Introduction

Per- and polyfluoroalkyl substances (PFAS) today include more than > 10,000 synthetic organofluorine chemical compounds with hydrophilic, lipophilic and surfactant properties^1^. Since their introduction in the 1950s, they have been broadly used amongst others in aqueous firefighting foam, cosmetics, textiles, carpets, coatings, plastics, and ski wax. Their chemical properties make many of them persistent organic pollutants that are potentially bioaccumulative, biomagnifying, and toxic^2^.

The environmental burden of PFAS has shown high spatial variation. PFAS can be emitted via point sources and locally deposited, while they also show long-range water and atmospheric transport or even translocation and dispersal via migratory wildlife, or a combination of multiple transport pathways^3,4^. For example, multiple PFAS have been emitted locally at firefighting training sites with further transport of the compounds to recipients and the atmosphere^2,3,5^. These transportation and dispersal mechanisms have contributed to PFAS nowadays being present ubiquitously in the environment.

Most studies on PFAS have been performed in freshwater and marine systems^6,7^. Our knowledge on the terrestrial fate of PFAS including transfer along terrestrial food chains and food webs is limited even if multiple terrestrial biota including earthworms^8^, birds^9^, rodents^9–11^ and their predators^12^, reindeer^13^, roe deer^14^, and polar bears^15^ have been studied. Plant-uptake, digestion of PFAS-coated soil particles, and uptake of PFAS-rich dust are all potential but poorly studied mechanisms contributing to PFAS also entering the terrestrial food chain with risk of bioaccumulation and biomagnification in consumers and predators.

In mammals, PFAS primarily accumulate in lungs, liver, kidney, and brain, which can be associated with PFAS being cancerogenic, causing chronic liver disease, and deteriorating endocrine, metabolic, and reproductive systems^16,17^. Accumulation of PFAS in liver is thought to induce liver lesions, and in a systematic review including 85 experimental rodent studies and 24 human epidemiological studies, Costello et al.^18^ concluded that PFAS consistently have induced steatosis and lipid accumulation in amongst others mice and rats (see also^19,20^). It is thought that PFAS promote liver inflammation mainly, but not exclusively, through an effect on peroxisome proliferator-activated receptor alpha (PPARα), causing an altered lipid metabolism^18^. The exact mechanism of liver toxicity remains however unresolved and there is still insufficient evidence to conclude that PFAS are hepatotoxic. In an experimental study, Blake et al.^21^ describe that PFAS treated mice showed cytoplasmic alteration that was characterized by varying degrees of hepatocellular hypertrophy that comprised decreased glycogen and intensely eosinophilic granular cytoplasm. Ultrastructural examination showed that the cytoplasmic alteration was associated with increases in mitochondria and peroxisomes, and vacuolation^21^. In their review, Thompson et al.^22^ concluded that steatosis is an inconsistent finding and that hepatocellular hypertrophy, increased mitoses and cellular death (including necrosis and apoptosis) are more typical. Importantly, toxicity studies on PFAS in terrestrial wildlife, especially non-predators are largely missing^7^.

In vitro assays have demonstrated PFAS-induced suppression of cytokine secretion by immune cells^23^. More specifically it is suggested that PFAS can reduce T-cell dependent IgM (Immunoglobulin M) antibody response (TDAR)^24^ and PFAS are overall suspected to immunocompromise organisms^25–27^. Hence, in wildlife, PFAS exposure could increase susceptibility to infectious pathogens, which to our knowledge has not been studied in natural settings.

Small mammals are keystone species for the functioning of boreal ecosystems. They consume mushrooms as well as plants and their berries, and contribute to the dispersal of cryptogams and vascular plants^28,29^. They are also important staple food for multiple mammalian and avian predators including Tengmalm’s owl (*Aegolius funereus*) that is specialized on voles^30,31^. The bank vole (*Clethrionomys glareolus*) is Europe’s most common mammal^32^. Even if the species is generally regarded as granivorous and herbivorous, it frequently also consumes mushrooms and invertebrates^33,34^. Due to their limited home ranges (up to ca. 0.4 ha)^35,36^, micropollutants detected in their tissue likely reflect local exposure (sensu^11,37^). Ungulates like roe deer (*Capreolus capreolus*) and moose (*Alces alces*) are browsers mainly feeding on twigs of trees and shrubs, but also feed on for example crops^38^ and consume mushrooms and berries (either deliberately or accidentally)^39^. The home range of roe deer is smaller (0.5 km^2^) than that of moose (ca. 72 km^2^^40^), but compared to bank voles, it is likely difficult to link uptake of pollutants in ungulates, especially those with large movement ranges, to potential point sources. The key role of bank voles in bottom-up and top-down ecosystem processes along with their feeding and movement ecology makes them a suitable model species to increase our understanding of the terrestrial fate of PFAS.

In previous studies of PFAS on the island Frösön at lake Storsjön, central Sweden, high PFAS concentrations were detected in groundwater (> 400,000 ng/L)^41^, and high levels in fish (Eurasian perch, *Perca fluviatilis*, PFOS (perfluorooctane sulfonate) mean concentrations at two sites: 27.5 and 41.1 ng/g)^42^ have resulted in the dietary recommendations to consume predatory fish including Eurasian perch not more than 2–3 times per year^43^. These findings pose the opportunity and need to study the fate including health effects of PFAS in the terrestrial food web on this island, hopefully expanding and deepening our general knowledge of the terrestrial fate of PFAS.

**Fig. 1:**
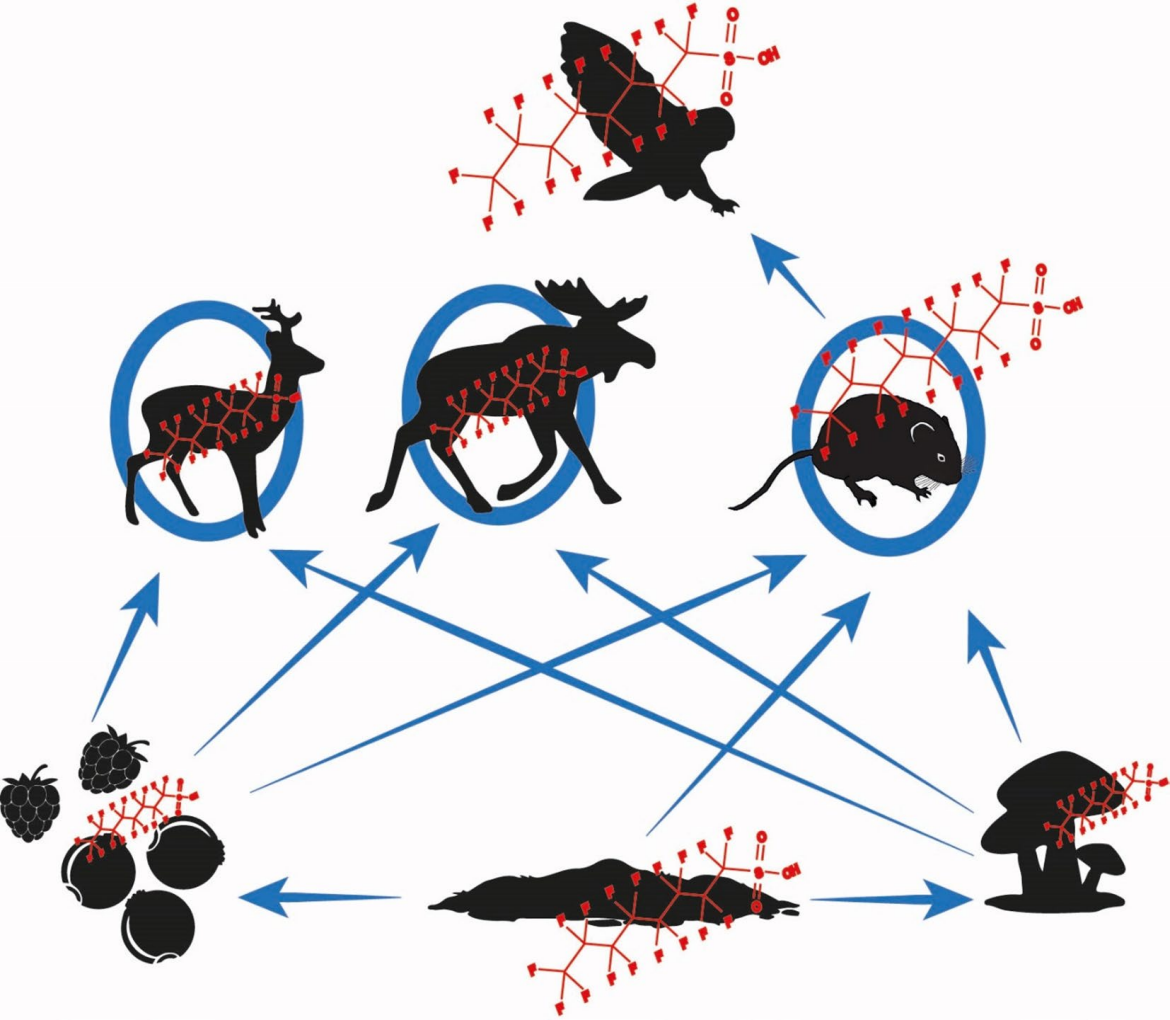
Expected trophic fate of PFAS (per- and polyfluoroalkyl substances). The projected bioaccumulation and biomagnification pathways of PFAS in the studied trophic network at Frösön, northern Sweden. PFAS in soil are likely taken up by dwarf-shrubs including lingonberry (*Vaccinium vitis-ideae*), blueberry (*V. myrtillus*), raspberry (*Rubus idaeus*), and mushrooms (here sweet tooth *Hydnum repandum*). Berries and mushrooms are important food for bank voles (*Clethrionomys glareolus*) and might also be eaten (either deliberately or accidentally) by ungulates including roe deer (*Capreolus capreolus*) and moose (*Alces alces*). Bank voles are staple food for the predator Tengmalm’s owl (*Aegolius funereus*).

We studied PFAS concentrations in soil and biota (mushrooms, berries of vascular plants, voles, ungulates, and owls), and health effects in bank voles. The latter focused on histopathological indicators and infection with *Orthohantavirus puumalaense* (PUUV), a common zoonotic pathogen in northern Sweden^44,45^. We predicted PFAS to accumulate in biota and to biomagnify along the food chains (Fig. 1). In the contaminated area characterized by high PFAS exposure, we hypothesized bank voles to demonstrate liver lesions and increased susceptibility to infection with PUUV.

## Results

### PFAS concentrations and profiles

**Fig. 2:**
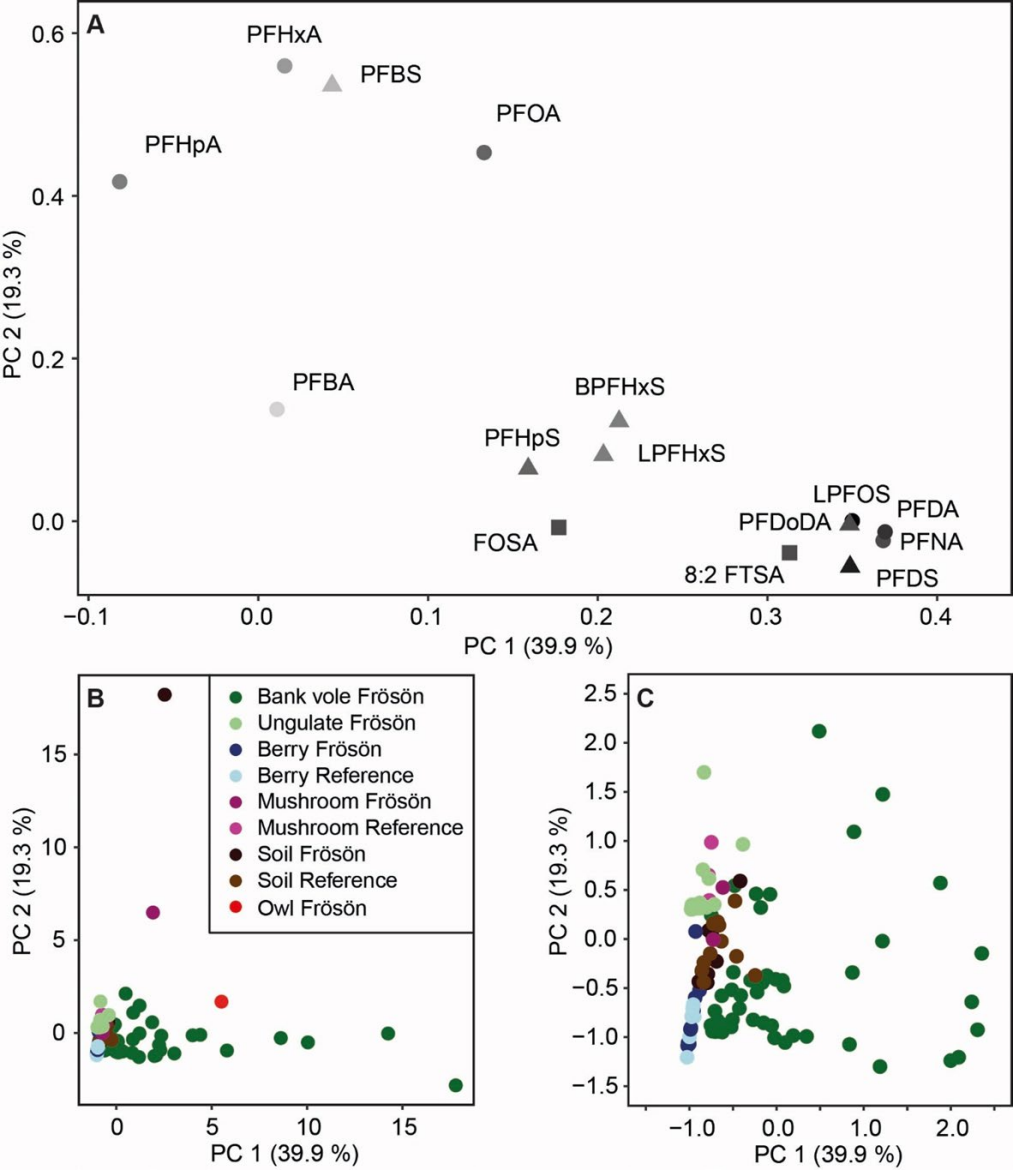
Multivariate analysis of PFAS (per- and polyfluoroalkyl substances) concentrations. Principal component analysis (PCA) on concentrations of 14 PFAS in environmental samples (soil, mushrooms, berries, ungulates, bank voles, and owl) on the island of Frösön and in the reference area. The PC loadings (**A**) illustrate the compositional similarities among the different PFAS concentrations, while in (**B**, **C**) the PC scores illustrate the similarities in PFAS concentrations among the different samples, with (**C**) representing a higher resolution of the PC scores < 2.5 for PC1 and PC2, respectively. Percentage of explained variation is given for the first two PCs. PFHxS is represented by its linear (L-PFHxS) and branched (B-PFHxS) isomers. In (**A**), PFCAs are represented by circles, PFSAs by triangles and PFAS precursors by quadrats with the length of the C chain illustrated by grey scale (light grey (C = 3) to black (C = 11)).

**Fig. 3:**
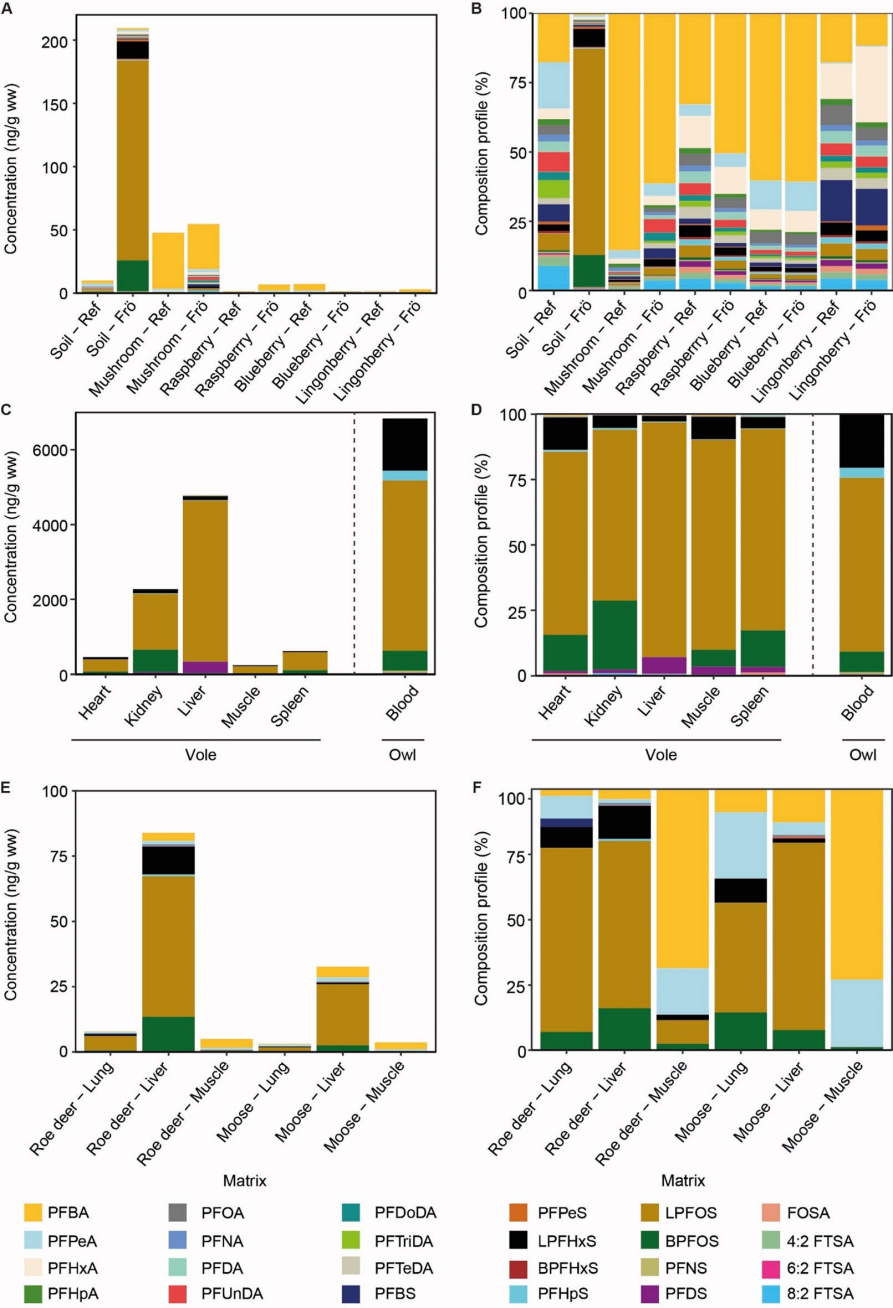
PFAS (per- and polyfluoroalkyl substances) concentrations and their profiles. Mean concentrations of PFAS (per- and polyfluoroalkyl substances) in (**A**) soil, mushrooms, berries, in the reference area (Ref; see also Supplementary Fig. S1) and on the island of Frösön (Frö), in (**C**) organs and tissue of bank voles and in blood of the owl, and in (**E**) organs of ungulates on Frösön. (**B**, **D**, **F**) represent the composition profile (relative concentration) of the studied PFAS. Please note that 6:2 FTSA was not analysed in soil, mushrooms and berries, PFNS and 4:2 FTSA not in voles, B-PFOS not in vole liver, and 4:2 FTSA and 6:2 FTSA not in ungulates. Bank voles and ungulates were only sampled on Frösön.

Of the 22 analysed PFAS, all were detected in at least two samples and considered for visualization and statistical analysis, and 15 PFAS were detected in all samples (see Supplementary Table S1). PFAS concentrations on Frösön showed large variations among the studied species, tissues, and organs (Figs. 2 and 3). Overall, our analyses revealed distinct patterns of concentrations of the different PFAS in the biota (Fig. 2). The first three PCs (principal components) explained 59% of all variation among PFAS concentrations. PC1 explained 40% of the variation, being dominated, in ascending importance, by 8:2 FTSA (fluorotelomer sulfonic acid), PFDoDA (perfluorododecanoic acid), PFDS (perfluorodecanesulfonic acid), linear (L)-PFOS, PFNA (perfluorononanoic acid) and PFDA (perfluorodecanoic acid), and PC2 explained 19%, being dominated by PFHpA (perfluoroheptanoic acid), PFOA (perfluorooctanoic acid), PFBS (perfluorobutane sulfonate), and PFHxA (perfluorohexanoic acid) (Fig. 2a). Bank vole samples were mainly associated with PC1 and their partly high PC scores indicated high PFAS concentrations (Figs. 2b and c and 3c). In addition, one soil, one mushroom and the owl sample from Frösön indicated high PFAS concentrations (Figs. 2b and 3a, Supplementary Table S1). Soil, mushrooms, berries and ungulates mainly separated along the PFAS gradient of PC2 (Fig. 2c).

These general patterns at Frösön and the reference area were confirmed by more detailed statistical analyses. The mean concentrations ± standard error (ng/g dry weight (dw)) of ƩPFAS at Frösön showed the following ascending pattern for soil, mushroom and berries: lingonberry (1.8 ± 1.2, *n =* 3) < raspberry (3.1 ± 1.1, *n =* 3) < blueberry (7.3 ± 1.9, *n =* 3) < sweet tooth (54.7 ± 20.6; *n =* 3) < < soil (196 ± 179, *n =* 10) (see also Fig. 3a, Supplementary Table S1). For the wildlife samples that were expressed in wet weight (ww), the mean concentrations ± standard error (ng/g ww) of the ƩPFAS showed the following pattern: lung (moose) (3.2 ± 0.5, *n =* 7) ≈ muscle (moose) (3.7 ± 0.7; *n =* 7) < roe deer (muscle) (5.0 ± 0.5, *n =* 9) < lung (roe deer) (7.9 ± 3.6, *n =* 10) < liver (moose) (55.4 ± 16.3, *n =* 8) < liver (roe deer) (84.0 ± 46,7; *n =* 10) < < muscle (bank vole) (238 ± 78.0, *n =* 12) < < heart (bank vole) (458 ± 130, *n =* 12) < < spleen (bank vole) (621 ± 210, *n =* 12) <<< kidney (bank vole) (2,280 ± 829, *n =* 12) <<< liver (bank vole) (4,790 ± 1,230, *n =* 12) (see also Fig. 3c, e, Supplementary Table S1). These differences in the concentration of the 22 PFAS among studied samples was also confirmed by two-sided ANOVA (Supplementary Table S2; see Fig. 3 for mean concentrations).

The concentrations of the linear (L-) and branched (B-) PFOS in soil were higher on Frösön compared with the reference area, while the concentrations of all other PFAS in either soil, berries or mushrooms did not differ between Frösön and reference area (for results from the *t*-tests, see Supplementary Table S3; see Fig. 3a for mean concentrations).

The PFAS profiles varied among samples and localities. In soil on Frösön, L-PFOS was on average the dominating PFAS (~ 74%), while PFAS in soil samples in the reference area were more evenly distributed with PFBA (perfluorobutanoic acid), PFPeA (perfluoropentanoic acid), and 8:2 FTSA contributing with a total of ~ 43% (Fig. 3b). On Frösön, the PFAS profiles in mushroom, raspberry and blueberry were dominated by PFBA, while PFHxA followed by PFBS and PFBA dominated in lingonberry (Fig. 3b). This pattern was similar in the reference samples, except that PFBA, although dominating, only constituted to ca. 1/3 of PFAS concentrations in raspberry, while PFBA made up > 50% of the PFAS on Frösön (Fig. 3b).

In bank voles, PFAS concentrations were highest in liver with L-PFOS being the dominating PFAS (> 65%) in all tissues and organs (Fig. 3c-d). Also, in roe deer and moose, PFAS concentrations were highest in liver and like in bank voles dominated by L-PFOS in liver and lung (Fig. 3e-f). However, muscle in the ungulates compared with bank vole were dominated by PFBA (Fig. 3c-f). The sum of PFAS was 6840 ng/g ww in the owl blood (Fig. 3c, Supplementary Table S1) and dominated by L-PFOS (Fig. 3d).

**Fig. 4:**
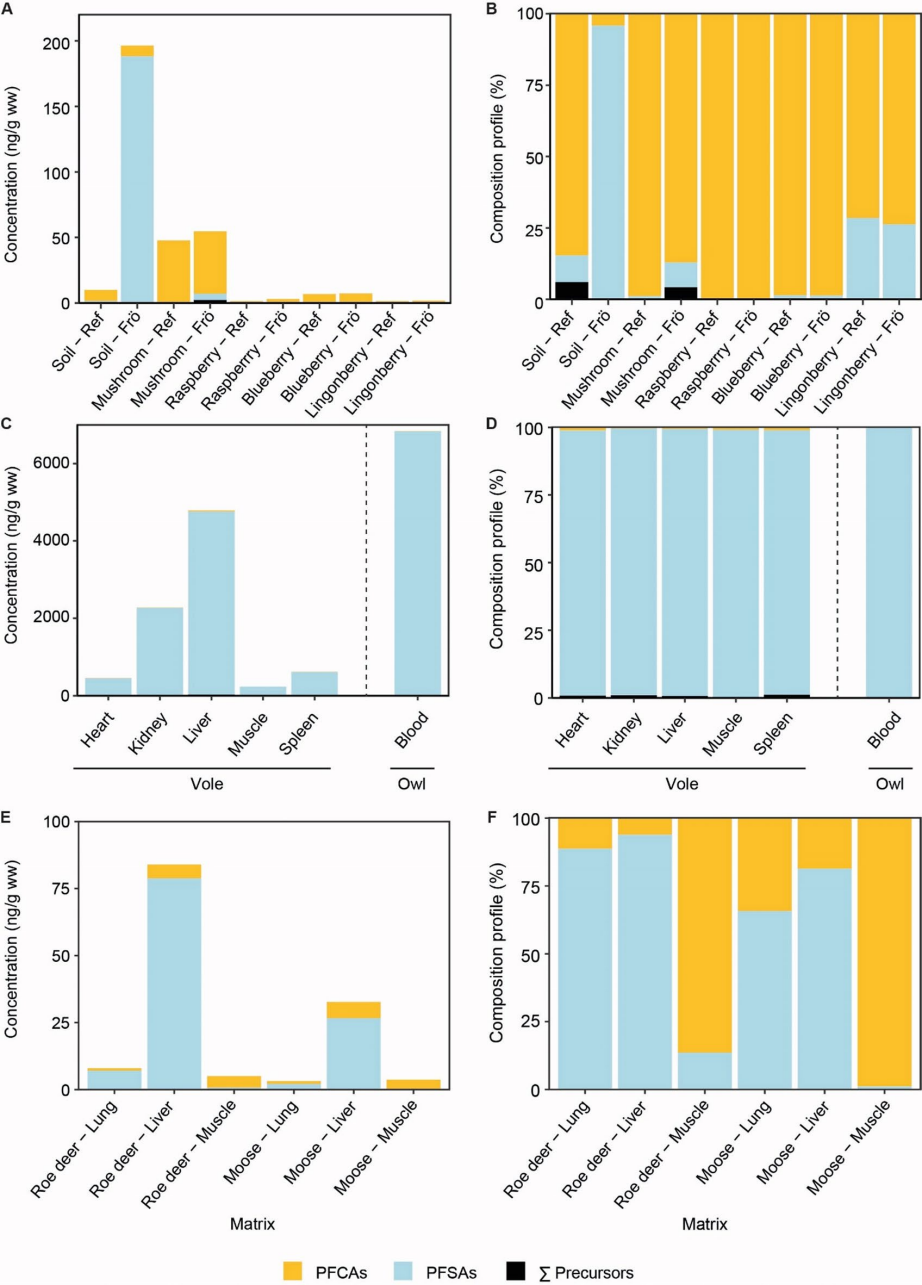
PFCA (perfluoroalkyl carboxylic acid), PFSA (perfluoroalkyl sulfonic acids) and the sum (Σ) of precursors concentrations and their composition. Mean concentrations in (**A**) soil, mushrooms, berries, and (**C**) organs and tissue of bank voles, owl blood as well as in (**E**) organs of the ungulates roe deer and moose in the reference area (Ref), and on the island of Frösön (Frö), northern Sweden. (**B**, **D**, **F**) represent the composition profile (relative concentration) of the studied PFAS. Please note that 6:2 FTSA was not analysed in soil, PFNS and 4:2 FTSA not in voles, B-PFOS not in vole liver, and 4:2 FTSA and 6:2 FTSA not in ungulates. Bank voles and ungulates were only sampled on Frösön.

Summarizing PFAS into PFCAs (perfluoroalkyl carboxylic acids), PFSAs (perfluoroalkyl sulfonic acids), and precursors revealed that the reference soil samples, as well as all mushroom and berry samples and ungulate muscles were dominated by PFCAs (Fig. 4). In contrast, soil samples on Frösön, and all vole samples, owl blood, ungulate lung samples and roe deer liver were dominated by PFSAs and the proportion of PFCAs and PFSAs was similar in moose liver (52% and 48%, respectively). (Figures 3c-f and 4).

The three roe deer with the highest PFAS4 (PFOA, PFNA, PFHxS (perfluorohexanesulfonic acid), PFOS) concentrations (> 45 ng/g ww) were hunted on the isthmus Bynäset or close to the isthmus, i.e., close to the firefighting training site on Bynäset (westernmost firefighting training site in Supplementary Fig. S1). It is also on this isthmus all bank voles were trapped and none of the vole pools had a PFAS4 concentration lower than 474 ng/g ww (max 11,600 ng/g ww) (Fig. 3c-d).

### Biomagnification of PFAS

**Fig. 5:**
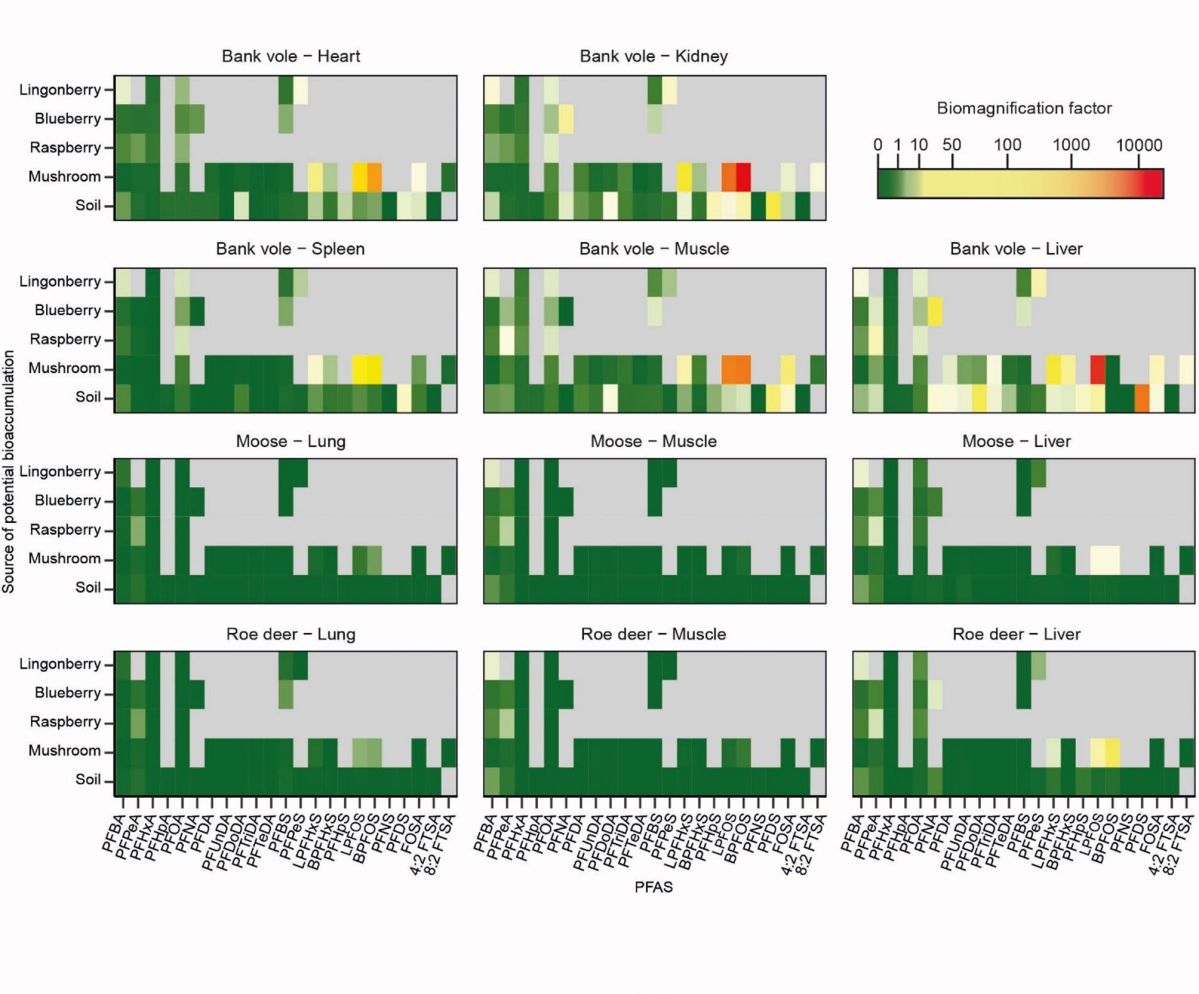
Biomagnification of PFAS (per- and polyfluoroalkyl substances). Biomagnification factors represented as a colour ramp for 21 PFAS (6:2 FTSA was excluded due to missing values in soil, mushrooms and berries) in different organs and tissue of bank voles, roe deer, and moose. The y-axis represents the potential food source of biomagnification. Soil represents a potential source that is accidentally taken up by species when foraging on the biota or when moving/digging in soil (voles). Grey areas represent cases with concentrations below limit of quantification at the lower trophic level, resulting in division by zero and white areas represent PFAS that were not analysed at one of the trophic levels.

**Fig. 6:**
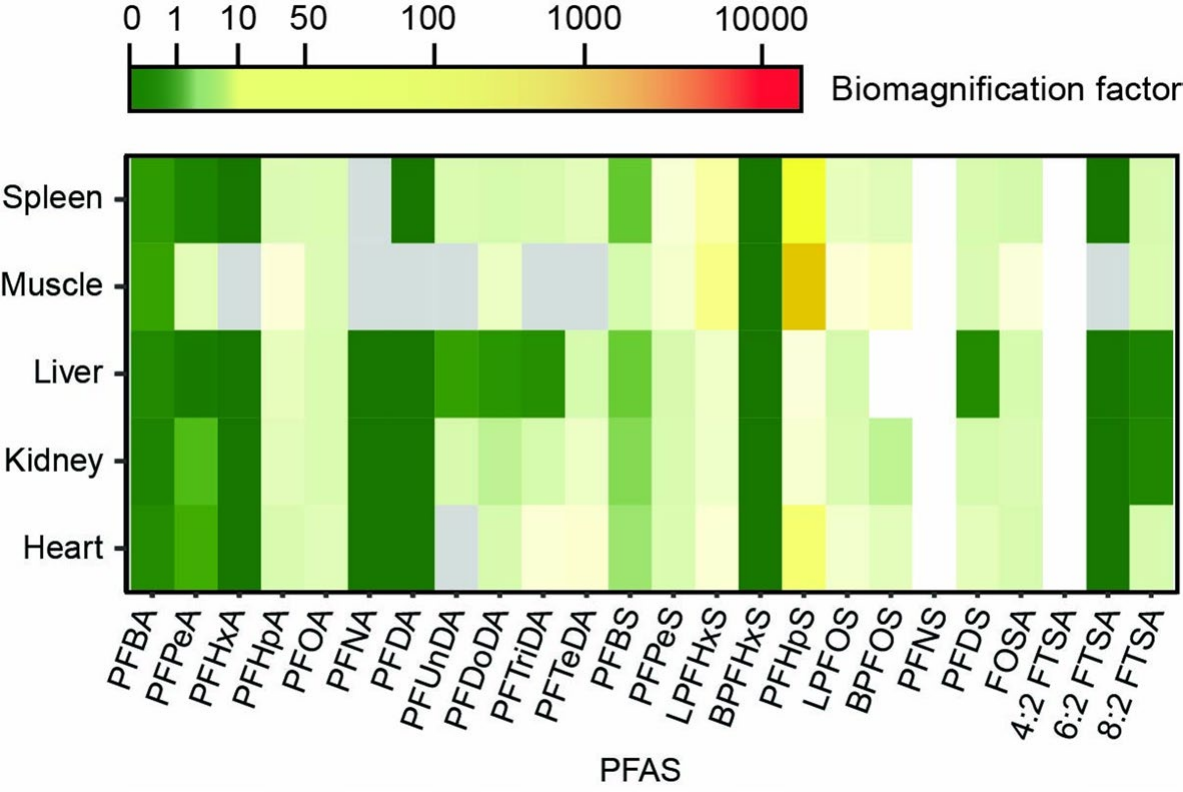
Biomagnification of PFAS (per- and polyfluoroalkyl substances) from voles (prey) to owls (predator). Biomagnification factors represented as a colour ramp for 22 PFAS in owl blood. The y-axis represents the potential food source of biomagnification represented by bank vole tissue and organs. Grey areas represent cases with concentrations below limit of quantification at the lower trophic level, resulting in division by zero and white areas represent PFAS that were not analysed at one of the trophic levels.

The biomagnification factors for several PFAS in bank voles were overall higher for mushrooms and soil than for berries and were > 100 in 27 out of 265 cases (10%) (Fig. 5, Supplementary Table S4). In contrast, the biomagnification factors for PFAS in the ungulates were overall low for soil but were > 1 for several PFAS originating from especially mushrooms and were even > 100 for L-PFOS and B-PFOS in a liver of a roe deer (Fig. 5, Supplementary Table S4). Overall, in ungulates, biomagnification factors were lower in lung and muscle than in liver, while in voles all studied tissues and organs showed biomagnification (BMF > 1) of several PFAS that originated from multiple sources (Fig. 5, Supplementary Table S4). Biomagnification in owl blood originated especially from PFHpS (perfluoroheptanesulfonic acid) in bank vole muscle, spleen, and heart and was > 1 for most PFAS (except PFBA and B-PFHxS) (Fig. 6).

### Health effects

The two pathologists agreed on histopathological changes in 21 and disagreed in 11 cases of the 32 studied bank voles. Where there was disagreement, pathologist no. 2 consistently scored the degree of changes one level higher than pathologist no. 1. Seven of the voles had minimal histopathological changes in the liver, meaning that no changes were observed that without doubt could be interpreted as deviations from the normal (Supplementary Table S5). An additional individual had minimal changes apart from multiple foci where several hepatocytes had 1–5 vacuoles in the cytoplasm (Supplementary Table S5). Two female and one male juveniles as well as two female and three male adults were among these animals with minimal changes. The main findings in the remaining animals were mild (*n =* 11), moderate (*n* = 3), marked (*n =* 6) and severe (*n =* 2) cytoplasmic alterations (Supplementary Table S5). The changes were diffuse, i.e., similar in all lobuli examined, but the location within the lobuli varied somewhat. The cells with cytoplasmic alteration did often show some degree of hepatocellular hypertrophy. A typical appearance was that the cytoplasm was hypereosinophilic and seemed uniformly finely granular or fragmented, what is described as “ground glass appearance”. The normal architecture was preserved, but the cell borders were often less distinct and cell nuclei less prominent than usual, and the sinusoids narrow or occluded by the enlarged hepatocytes. In addition, some animals showed mild (*n =* 5) or moderate (*n =* 5) cytoplasmic vacuolation, typically with few and small vacuoles in some hepatocytes, either dispersed or in small areas (Supplementary Table S5). Dead hepatocytes (necrotic or apoptotic) and degenerated cells were observed, but not in large numbers. Binucleate cells were common but interpreted as an incidental finding. Hyperplasia was not observed, and inflammation was only observed as an incidental finding of mild mononuclear cholangitis (Supplementary Table S5).

There was no clear association between sex, age, and PFAS concentrations in bank vole liver and between PFAS concentrations in bank vole liver and observed cytoplasmatic changes with concentrations of PFBA and PFPeS (perfluoropentanesulfonic acid) however being higher in juveniles than in adults (Table 1; Supplementary Table S6). The vole pool with the second highest L-PFOS concentrations in liver contained a bank vole with only mild cytoplasmatic changes and one of the bank voles with severe diffuse panlobular granulation (score 5) belonged to a pool with the third lowest L-PFOS concentrations (but still > 1200 ng/g ww) (Table 1). PFAS concentrations in vole pools did not differ between the groups of minimum cytoplasmatic change (change = 1 or 2) and among the groups of maximum cytoplasmatic change (change = 2–5) per pool (Supplementary Table S6). The correlation between PFAS and cytoplasmatic change was significant for 1–16% of all potential combinations of PFAS concentrations and cytoplasmatic change (Supplementary Table S6).

**Table 1:** Characteristics of the studied bank vole pools. The 12 pools of in total 32 bank voles studied for PFAS (per- and polyfluoroalkyl substances) and liver histopathology showing the sex (F female, M male), age (J juvenile, A adult), number of voles per pool (N), concentrations (ng/g ww) in liver of four PFAS that have been previously studied for toxic effects, and assessment (mean based on two independent assessments; range with a maximum of 1–5 (mild-severe) is given in parentheses) of observed cytoplasmatic changes. Liver sections for all 32 bank voles along with detailed descriptions of histopathological observations are given in supplementary table S5.

Pool	Sex	Age	*N*	PFOA	PFNA	L-PFOS	PFHxS	Cytoplasmatic changes
1	F	J	3	2.2	7.3	4430	285.7	1.8 (1–3)
2	F	J	3	0.3	2.4	3086	71.4	3.2 (2–4)
3	F	J	3	1.4	3.2	3073	68.8	3.5 (2–4)
4	F	A	3	0.3	0.5	469	5.5	1.8 (1–3)
5	F	A	2	1.1	10.4	10,749	252.0	2.0 (1–3)
6	M	J	3	1.3	3.1	2736	125.4	2.5 (1–5)
7	M	J	3	1.1	2.5	1371	7.2	2.5 (2–3)
8	M	J	2	1.4	7.5	11,380	178.1	2.0 (2–2)
9	M	J	2	0.8	3.6	8957	149.0	3.0 (2–4)
10	M	A	3	0.3	0.5	2903	26.42	3.0 (1.5-5)
11	M	A	3	0.8	1.4	1270	79.32	2.8 (1–5)
12	M	A	2	1.4	0.5	1222	13.22	2.5 (1–4)

Five of the 19 susceptible bank voles (26.3%) tested positive for infection with the zoonotic pathogen (PUUV). Nine of the 12 vole pools included bank voles susceptible for PUUV infection and four of the nine pools included both PUUV positive and negative individuals with the remaining five pools testing PUUV negative (Supplementary Table S7). Therefore, we could not meaningfully analyse the association between infection and PFAS liver concentrations. Mean cytoplasmatic changes did not differ between infected and non-infected bank voles (W_14, 5_ = 43.5, *p* = 0.452).

## Discussion

Our study reveals extremely high concentrations of PFOS in terrestrial biota and provides valuable insights into the trophic fate of PFAS from soil via mushrooms and berries to rodent and ungulate herbivores and a specialized vole predator. Importantly, the observed histopathological changes, despite absence of statistical link to PFAS concentrations, along with the exceptionally high PFOS concentrations and pathogen prevalence suggest potential health effects in wildlife that are in line with predictions based on laboratory studies.

PFAS have immunosuppressive properties in humans and other mammals^25,26^ and are associated with increased disease risk in already low-exposed human populations^46^. The high PFAS concentrations in bank voles are therefore a potential health risk for the specimens and could induce increased susceptibility to infection with pathogens. Indeed, the prevalence of PUUV that we measured in the bank voles (26.3%), but based on a moderate sample size (*n*_*Seasons*_ = 1, *n =* 19 individuals) and a slightly different trapping design, is the third highest measured during late summer/early autumn in the endemic area of PUUV in Sweden (compared with data (*n*_*Seasons*_ = 36, *n* = 6,233 individuals) from available periods 1979–1986 and 2003-2013^44^ and compared with unpublished data (*n*_*Seasons*_ = 83, *n* = 11,754 individuals) for the whole period 1979–2020). Neither our histopathological nor the pathogen analyses reflected PFAS concentrations in the vole pools. This might indicate that the here studied PFAS concentrations in liver (lowest PFOS concentrations 469 ng/g ww), compared to those measured in 2017–2018 in bank voles 100 km west of the study area (mean 2.4 ng/g ww)^11 ^were that extreme that they confounded any detectible relationship between PFAS and health of the voles.

Experimental studies are needed to confirm if PFAS indeed increases susceptibility for infection with PUUV or other epizootic or zoonotic pathogens. PUUV causes the zoonotic disease *nephropathia epidemica* (vole fever) in humans^44,47–50^. If PFAS increase susceptibility for infection in bank voles, these compounds could potentially also pose an indirect threat to human health via infectious disease.

The non-difference of PFAS concentrations in mushroom and berries between Frösön and the reference area was surprising. This might either suggest that mushrooms and the studied plant species do not accumulate PFAS (but are taken up; see below) or that also the reference area was impacted by PFAS originating from diffuse sources. As expected, PFAS concentrations were highest in liver of bank vole, roe deer and moose, which is in line with previous findings in rodents^9,18^ and ungulates^14^. The high PFOS concentrations that we found in bank voles are of similar magnitude (469 − 11,380 ng/g ww) as those found in rodents (24–97,000 ng/g ww) in a desert oasis in the United States that is affected by firefighting foam used at a firefighting training site^9^. The total PFOS concentrations in our bank vole livers were likely even higher than reported here. Liver results included only the linear (L-PFOS) and not the branched isomer (B-PFOS), with B-PFOS in the bank vole kidneys representing 14–35% of total PFOS concentrations. Despite rather high concentrations of PFOS in soil, this PFAS was not accumulated in berries, while the mushroom sweet tooth showed high affinity for PFOS, with the latter being supported by previous studies on oyster mushroom (*Pleurotus ostreatus*)^51^. In plants, uptake and transportation of PFAS from roots to fruits is limited by the chain length of the PFAS with short-chained compounds (C < 7) being highly water soluble and hence more mobile than long-chained PFAS like PFOS (C = 8)^52,53^, which explains the comparatively low PFOS concentrations in the studied berries. This PFOS behaviour might also explain the observed differences in PFOS concentrations among the herbivores. Roe deer and moose are browsers feeding largely on twigs^38^. In addition, and in contrast to bank voles, they have large home ranges^40 ^with feeding over large areas likely diluting any PFAS signal from point sources.

The high biomagnification factors in bank voles for several PFAS originating in soil and/or mushroom suggest biomagnification, which is also supported by similar PFAS profiles of soil on Frösön and bank vole tissue and organs. These findings are further supported by the feeding ecology of bank voles. The voles frequently feed on berries of raspberry, blueberry, and lingonberry^33,34,54^. This is likely also the case in our study, and the rather low PFAS concentrations in berries in the present study did not result in biomagnification in bank voles. Also mushrooms are important food for bank voles^33 ^which in our study might explain mushroom-dependent biomagnification of PFAS in bank voles. However, the PFAS profiles in mushrooms differed from those in bank voles, which suggests that also other food or other mushrooms might be involved in the biomagnification of PFAS in the food web on the island Frösön. We did not study the gut content of the bank voles. Therefore, we do not know their actual food, and whether the here studied potential PFAS sources (soil, mushrooms, berries) are the actual sources of the high PFAS concentrations in bank voles. The calculated biomagnification factors and PFAS profiles strongly suggest soil to be an important PFAS source for bank voles. Despite bank voles being classified as herbivores, they also consume invertebrates, and mineral particles are commonly found in their stomach^34,54–56^. In fact, future studies should include the role of macroinvertebrates in general and earthworms in particular for bioaccumulation of PFAS in bank voles sensu Grønnestad et al.^10^. Bank voles dig their own burrows and dust formed during digging, resulting in either inhaling or digestion of PFAS-coated dust particles, could be an important PFAS source.

The bank vole is Europe’s most common mammal^32^ and important staple food for many mammalian and avian predators^31,57,58^ with Tengmalm’s owl depending to up to 90% on bank voles as prey species^31,59^. The PFAS (especially L-PFOS) concentrations and profiles in the owl blood along with the biomagnification factors (especially for PFHpS) based on PFAS concentrations in vole tissue and organs support biomagnification from the staple food to the predator. In a previous study area free of point sources 100 km west of the present area, the mean PFAS concentrations in owl blood were ca. 20 ng/g ww^60 ^i.e., in the present study they were two magnitudes or ca. 340 times higher (6840 ng/g ww). In rodents, the serum elimination half-live of PFOS is ca. 1–2 months^61^. The juvenile owls sampled in our study were ca. 2–3 weeks old when sampled. While the PFAS source in owl blood is vole food, we do not know if the high PFOS concentrations in blood were a result of maternal transfer via eggs that have higher PFOS concentrations than blood^60^ or a result of feeding on voles after hatching. While PFAS accumulate in liver, they are initially absorbed in blood during a depuration period^62^. The observed high PFOS concentrations could therefore be even higher in owl tissue including liver. We do not know where the owl parents were hunting. Given the ca. 200 ha home range of Tengmalm’s owl^63,64 ^it is reasonable to assume that they were hunting for voles in forests near one of the three firefighting training sites on the island (distance from nest box 400, 1900 and 2100 m, respectively; Supplementary Fig. S1). Predators generally have broader home ranges than their prey. When hunting on PFAS-loaded bank voles that have a home range of up to ca. 0.4 ha^35,36 ^Tengmalm’s owls that also are nomadic^65^ can translocate and disperse PFAS in the landscape, and thereby contribute (either via food or their own PFAS burden) to secondary distribution of PFAS in the environment^4^.

The identified PFAS concentrations in soil, sweet tooth, and wildlife – especially in bank vole and Tengmalm’s owl – are worrisome from the perspective of ecosystem, wildlife and human health. The histological examination of vole livers revealed that many of them had changes that can be described as diffuse cytoplasmic alteration and hepatocellular hypertrophy. Such changes may indicate that the cytoplasmic volume is increased due to microsomal enzyme induction or peroxisome proliferation^66^. This is consistent with descriptions of histological changes in mice experimentally exposed to PFOA and hexafluoropropylene oxide dimer acid^21^. It is hence plausible that these changes are associated with the extremely high concentrations of PFAS, despite no voles from control areas being available for validation. Due to low tissue amount per vole, we had to pool vole samples from 2 to 3 voles for PFAS analyses. This unfortunately hindered us from relating histopathological results of individual voles to individual PFAS concentrations. The range (up to 1–5 per pool) of cytoplasmatic changes observed in pools could therefore be explained by high potential variation of PFAS concentrations among voles. Indeed, also Witt et al. 2024^9^ observed high range of for example PFOS concentrations (24–97,000 ng/g ww) in white-footed mice (*Peromyscus leucopus*) in a PFAS-affected study area. Future studies relating environmental contaminants to health status should therefore preferably be done on specimen-based samples from both reference and contaminated sites.

Our study is also relevant from a food safety perspective. The maximum levels for PFOS, PFOA, PFNA, PFHxS, and their sum (PFAS4) in meat (5.0, 3.5, 1.5, 0.6, and 9.0 ng/g ww, respectively) set by the European Commission^67^ were not exceeded in any of the samples. However, the muscle of one roe deer was close to the threshold level of 5.0 ng/g ww for PFOS (4.3 ng/g ww). The maximum level for PFOS in offal (50 ng/g ww)^53^ were exceeded in liver of two roe deer.

Mountain hare (*Lepus timidus*) is a common and popular game species in northern Sweden that was not included in our study. Ecologically, mountain hares resemble bank voles including exposure to soil and foraging ecology. The high PFAS concentrations found in bank voles therefore urge to gain knowledge on PFAS in mountain hare, which is also important from a food safety perspective.

To our knowledge, this is the first attempt to study PFAS-induced histopathological and health effects in a terrestrial wildlife species. Our correlative study that suffered from the unfortunate need to pool vole samples and lack of samples from reference sites, needs to be followed up by replicated experiments that unravel the (foraging) behavioural and physiological mechanisms that induce liver damage and infection susceptibility, with the latter preferably be tested with pathogens known to cause stress-triggered infections.

## Methods

### Field sampling

We performed our study on the island of Frösön (41.6 km^2^; 63°11’N, 14°32’E) at lake Storsjön, boreal central Sweden, that has been identified as a hotspot of PFAS contamination in Sweden^41,42^ (Supplementary Fig. S1). As a reference, we used samples near Umeå, ca. 300 km north-east of Frösön (Supplementary Fig. S1). The reference sites were free from known point sources, but a most recent study detected PFAS in terrestrial wildlife in that region (ca. 35 km from sampling sites used in present study)^60^. To reflect potential exposure to and bioaccumulation and biomagnification of PFAS, we sampled soil at Frösön (Frö) and reference sites (Ref) (*n*_*Frö*_ *=* 10 and *n*_*Ref*_ *=* 11 pooled samples), berries of dwarf-shrubs lingonberry (*Vaccinium vitis-ideae*) (*n*_*Frö*_ *=* 3 and *n*_*Ref*_ *=* 3 pooled samples), blueberry (*V. myrtillus*) (*n*_*Frö*_ *=* 3 and *n*_*Ref*_ *=* 3 pooled samples), and raspberry (*Rubus idaeus*) (*n*_*Frö*_ *=* 3 and *n*_*Ref*_ *=* 3 pooled samples), and the edible mushroom sweet tooth (*Hydnum repandum*) (*n*_*Frö*_ *=* 3 and *n*_*Ref*_ *=* 3 pooled samples). According to the Right of Public Access (Allemansrätten), we did not need any permits to pick the berries and mushrooms. The species were identified and sampled by Åke Nordström in August-September 2022 and no voucher specimens of the material has been deposited. Soil samples of ca. 50 g were taken from the organic soil layer (O-horizon) and put in plastic bags. Berries from 40 to 80 plants/clones were sampled to generate pooled samples of ca. 200 g wet weight (ww). Pooled samples for mushrooms comprised 4–5 fruitbodies to generate 200–250 g ww. Localities had an inter-distance of at least 100 m.

In addition, at Frösön, we sampled tissues and organs of bank voles (*n =* 32 specimens), roe deer (*n =* 10) and moose (*n* = 8). Bank voles were snap-trapped opportunistically in forested areas during three trapping sessions (13–14 July, 11–12 August and 13–14 September 2022) during the peak phase of the vole cycle in the region^68^ using dried apples as bait. The voles died immediately upon trapping as indicated by all of them getting trapped over the neck. Bank voles were trapped at three sites with an inter-distance of at least 100 m (for coordinates see Supplementary Table S7). The traps were visited daily with an interval of ca. 8 h. The average daily ambient temperature during the trapping sessions was 11.2 (min-max 10-12.4), 14.6 (14-15.1), and 10.9 °C (10.2–11.5), respectively (weather station Östersund-Frösön Flygplats, smhi.se). Already in the field, all trapped bank voles were dissected, and muscle, liver, lung, heart, kidney, and spleen were extracted. Of the bank voles, 18 were males (10 juveniles and eight adults) and 14 females (nine juveniles and five adults). A liver subsample consisting of a slice of approximately 3 mm thickness cut in a frontal plane respective to the longitudinal axis of the animal was put in a tube with 10% neutral buffered formalin for histopathological analyses, while all other samples were stored in a portable freezer (-20 °C) during fieldwork and stored at -20 °C until further processing in the laboratory. The tissues and organs except lungs of the 32 bank voles were, prior to laboratory analyses, pooled into 12 samples in a stratified-random process. First, voles were classified into functional groups based on sex (male or female), and age estimates based on weight and signs of sexual maturity (juvenile or adult), resulting in four functional groups: female juveniles, female adults, male juveniles, and male adults. Individual vole samples per tissue and organ and per functional group were then randomly assigned to one of the 12 pools with 2–3 specimens per pool, resulting in three pools of female juveniles, two pools of female adults, four pools of male juveniles, and three pools of male adults. To study potential biomagnification of PFAS from bank voles to a specialized predator on Frösön, we also set up 10 nest boxes for Tengmalm’s owl (for more details see Ecke et al.^60^). There were unfortunately no breeding Tengmalm’s owls in the nest boxes the year when we trapped voles. However, we kept the nest boxes and checked them again in spring 2025. There was one breeding pair with three juveniles and we took a pooled blood sample (not more than 1 mL per juvenile) 25 April 2025 from the ca- 2–3 weeks old juveniles and analysed it for PFAS. We pooled the blood to increase sample volume for the PFAS analysis, which was also motivated considering that the brood shares food. No blood samples were taken from the adult owls since they are nomadic^65^ and any PFAS concentrations and profiles would not reflect the local and landscape conditions.

Samples of roe deer and moose on Frösön were provided by local hunting teams during the hunting season 18 September 2021–21 January 2022 (see Supplementary Table S7 for details on individual samples). Hunters followed a standard protocol and provided ca. 1 cm^3^ samples of muscle, lung, and liver that they put in plastic zip-bags and stored at -20 °C in the premises of the municipality of Östersund. After the hunting season, samples were transported on icepacks to the laboratory.

The sampling of small mammals was approved by the Animal Ethics Committee in Umeå (A 18-2019) and by the Swedish Environmental Protection Agency (NV-07483-19). All applicable institutional and national guidelines for the use of animals were followed. We complied with the ARRIVE guidelines.

### Histopathological analyses of bank vole livers

**Fig. 7:**
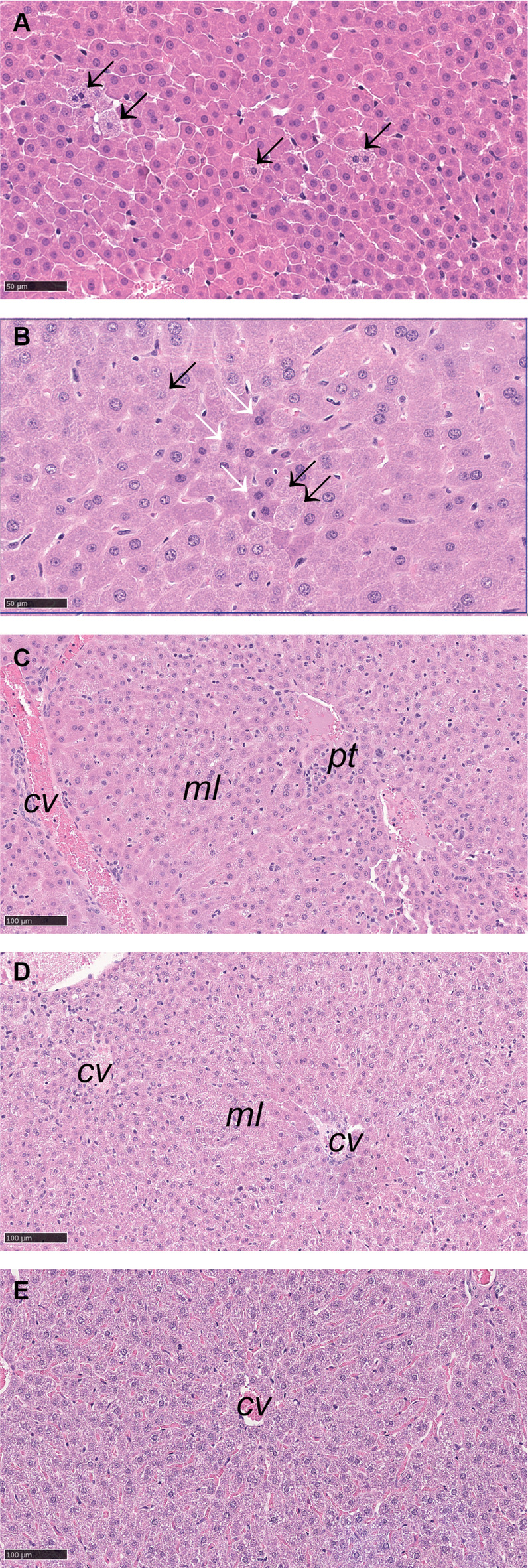
Examples of observed cytoplasmatic changes. Sections of bank vole livers representing (**A**) minimal changes characterized by hepatocytes of normal appearance with occasional randomly scattered hepatocytes with mild granulation (arrows) (score 1; bank vole 17, 400× magnification), (**B**) mild changes characterized by diffuse mild hepatocellular hypertropy and diffuse midlobular granulation – image showing mixture of hepatocytes with mild cytoplasmic granulation (black arrows) and hepatocytes with more normal appearance (white arrows) (score 2; bank vole 6, 400×), (**C**) moderate changes characterized by diffuse cytoplasmic granulation of hepatocytes in the midlobular zone (ml) between the central vein (cv) and the portal triade (pt) (score 3; bank vole 32, 200×), (**D**) marked diffuse cytoplasmic granulation of hepatocytes in the midlobular zone (ml) and possibly also the perilobular zone, while the centrolobular hepatocytes surrounding the central vein (cv) have more normal appearance (score 4; bank vole 30, 200×), and (**E**) severe diffuse panlobular granulation (score 5; bank vole 1, 200×). Tissue sections of bank vole livers were stained with haematoxylin-eosin.

The formalin-fixed liver samples were routinely processed for histology by dehydration in alcohol and xylene and embedding in paraffin. The paraffinized tissue was cut into 5–6 μm-thick sections and stained with hematoxylin and eosin according to standard procedures. The sections were examined by light-microscopy by two trained veterinary pathologists (BY and AL). Histopathological findings were classified based on the liver nomenclature guidelines recommended by the International Harmonization of Nomenclature and Diagnostic Criteria (INHAND) documents published in Toxicological Pathology^66^. Hepatocytes were regarded as necrotic only if dying cells or debris were surrounded by inflammatory cells, and not based on changes in single hepatocytes alone. The histological features of an animal may vary depending on multiple factors including food, and stress level. As we did not concurrently harvest livers from control voles, i.e., age- and gender-matched bank voles living in comparable areas but not exposed to PFAS, we chose to rely on comparison between individual livers. The following histopathological phenomena were scored on a scale from 1 to 5 (minimal-mild-moderate-marked-severe) according to the descriptions given by Thoolen et al.^66^ (see also Fig. 7): Cytoplasmic vacuolation, pigment deposition, cytoplasmic alteration, hepatocellular hypertrophy, hepatocellular hyperplasia, degeneration, cellular death, karyocytomegaly, multinucleated hepatocytes, inflammation, and potential other deviations. All observations were described according to distribution in the liver (focal – multifocal – diffuse) and location within the liver lobules (peri-, mid-, centro- or panlobular). Two pathologists (BY and AL) scored the histopathological characteristics and upon disagreement, the mean score was used.

### Analysis of orthohantavirus puumalaense

In its endemic area, PUUV, family Hantaviridae, order Elliovirales, is common in its only known reservoir host, viz. the bank vole^69^. In humans, PUUV causes a mild form of hemorrhagic fever with renal syndrome known as nephropathia epidemica^69^. In Sweden, our study area is situated in one of the four northernmost counties in Sweden where PUUV is endemic^44,70^. We analysed lung samples from bank voles by enzyme-linked immunosorbent assay (ELISA) to detect anti-PUUV IgG antibodies and identify zero-positive individuals, as described previously^49,71 ^except that we used another secondary antibody, i.e. an anti-mouse IgG (whole molecule) − alkaline phosphatase antibody produced in goat (cat. no. A5153, lot no. SLCJ8310, Sigma-Aldrich, Merck KGaA, Darmstadt, Germany). The antibody was validated as described earlier^66^.

We calculated the prevalence, i.e., the percentage of PUUV-infected voles, based on specimens susceptible for infection (≥ 14.4 g) since young bank voles (< 14.4 g) are protected from infection due to maternal antibodies^45,72^. Any potential spatio-temporal association between PFAS exposure and PUUV infection in bank voles seems plausible, considering the small home range of bank voles (up to ca. 0.4 ha)^35,36^, their short lifetime (majority not becoming older than 1 year)^73^, PFAS-induced immunosuppression^25,26^, and that zero-positivity represents an ongoing infection in bank voles since shedding of PUUV is life-long^74^.

### Chemicals for chemical analyses

In total, 22 target PFAS were included namely C_3_-C_13_ PFCA (PFBA, PFPeA, PFHxA, PFHpA, PFOA, PFNA, PFDA, PFUnDA (perfluoroundecanoic acid), PFDoDA, PFTriDA (perfluorotridecanoic acid), PFTeDA (perfluorotetradecanoic acid)), C_4_-C_10_ PFSA (PFBS, PFPeS, PFHxS, PFHpS, PFOS, PFNS (perfluorononanesulfonic acid), PFDS), 4:2, 6:2 and 8:2 fluorotelomer sulfonates (4:2 FTSA, 6:2 FTSA, 8:2 FTSA), and perfluorooctane sulfonamide (FOSA). For PFHxS and PFOS the linear and branched isomers were quantified separately (L-PFHxS, B-PFHxS, L-PFOS, and B-PFOS, respectively). In addition, 17 mass-labeled internal standards (IS) were used, which were spiked to the samples before extraction (Wellington Laboratories):^13^C_4_-PFBA, ^13^C_5_-PFPeA, ^13^C_5_-PFHxA, ^13^C_4_-PFHpA, ^13^C_8_-PFOA, ^13^C_9_-PFNA, ^13^C_6_-PFDA, ^13^C_7_-PFUnDA, ^13^C_3_-PFDoDA, ^13^C_2_-PFTeDA, ^13^C_3_-PFBS, ^13^C_3_-PFHxS, ^13^C_8_-PFOS, ^13^C_2_-4:2 FTSA, ^13^C_2_-6:2 FTSA, ^13^C_2_-8:2 FTSA, ^13^C_8_-FOSA.

### Sample Preparation for chemical analyses

All tissue biota samples except bank vole lungs that were used for pathogen analyses, were extracted using a sample aliquot of approximately 0.5 g homogenized tissue in a Precellys^®^ Evolution vials and subsequently 3 mL acetonitrile and PFAS IS mixture was added (100 µL of 0.05 µg/mL for individual PFAS). The samples were homogenized and extracted using Precellys^®^ Evolution, setting the parameters as 5000 rpm, 2 × 40 s, 20 s break in between. Subsequently, the Precellys^®^ Evolution vials were sonicated for 30 min and then centrifuged (3000 rpm for 5 min) and the supernatant transferred into a new 15 mL polypropylene (PP) tube. The sonication extraction was repeated with 3 mL acetonitrile for 30 min and centrifugation (3000 rpm for 5 min) and the supernatant was transferred into the 15 mL PP tubes. The 15 mL tubes were placed into a freezer (-20 °C) for > 16 h to crush the proteins. Thereafter, the PP-tubes were centrifugated at 4000 rpm at -5 °C for 15 min and the supernatant was transferred into new PP-tubes. Then, the supernatants were concentrated to 1 mL under a gentle steam of nitrogen. For clean-up, the 1 mL extracts were transferred into 1.7 mL Eppendorf centrifuge tubes containing 25 mg ENVI-Carb and 50 µL glacial acetic acid. After vortexing for 30 s, the Eppendorf centrifuge tubes were centrifuged at 4000 rpm at -5 °C for 15 min. Finally, the supernatants were transferred into 1.5 mL PP-vials and stored in a freezer (-20 °C) before analysis.

The soil, mushroom and berry samples were first freeze-dried until the samples were dry (3–7 days). Approximately 1 g dw sample aliquots were weighted into 15 mL PP-tubes. Then, the samples were spiked with an IS mixture (50 µL of 0.05 µg/mL for individual IS) and 3 mL methanol were added and sonicated for 30 min. After centrifugation at 3000 rpm for 15 min, the supernatants were transferred into another 15 mL PP-tube. The extraction was repeated twice with 3 mL methanol with 20 min sonication. The combined extracts were run through an ENVI carb cartridge (1 g, 12 cc) and collected in clean PP-tubes, and then air was pressed through the cartridge using a syringe. Next, the extracts were concentrated to 100 µL and transferred to a 1.5 mL PP vial. Finally, 400 µL methanol was added, vortexed and vials were stored in a freezer (-20 °C) before analysis^75^.

For quality control, blank samples were prepared for all batches (*n* = 10) and extracted in the same manner as the natural samples, but without any sample material.

### Chemical analyses

Instrumental analysis was performed using ultra-high pressure liquid-chromatography (SCIEX ExionLC AC system) coupled to a tandem mass spectrometry (SCIEX Triple Quad™ 3500) (UHPLC-MS/MS). The column oven was set to 40 °C, and 10 µL of sample were injected into a Phenomenex Kinetex C18 (30 × 2.1 mm, 1.7 μm) precolumn coupled to a Phenomenex Gemini C18 (50 mm × 2 mm, 3 μm) analytical column for chromatographic separation. The mobile phase consisted of MilliQ water with 10 mM ammonium acetate and MeOH. Data evaluation was performed using SciexOS software (2.0) (for details see^75)^.

The limits of detection (LOD) and limits of quantification (LOQ) were calculated based on the blank concentrations as follows:

$$\begin{gathered} {\text{LOD}}=\left[ {{\text{mean concentration in the blank}}} \right]+3 \times {\text{standard deviation}} \hfill \\ {\text{LOQ}}=\left[ {{\text{mean concentration in the blank}}} \right]+10 \times {\text{standard deviation}} \hfill \\ \end{gathered}$$

If no PFAS were detected in the blank samples, the LOD and LOQ were determined from the lowest point of the calibration curve used in data analysis, based on a signal-to-noise ratio of at least 3 and 10, respectively (Supplementary Table S8). Recoveries of individual PFAS in tissue and soil samples were, on average, 82 ± 11% and 91 ± 18% (Supplementary Table S8).

### Data conversion from fresh weight to dry weight

PFAS in soil, mushrooms and berries were based on freeze-dried samples and hence expressed as concentration in ng/g dw. To enable comparison with the wildlife samples that were expressed in ng/g wet weight, we calculated conversion factors from wet weight to dry weight. Previous studies have shown that PFAS preferentially distribute in liver, kidney and blood due to the PFAS binding to blood serum proteins und thus the accumulation of PFAS in fat tissue is low^76^. For the deer and moose samples, we used 0.5–1.4 g wet weight from the samples of the 18 specimens that were included in our study, freeze-dried them (typically 5 days and up to 7 days until samples were dry) and calculated the water content.

For the vole samples, we unfortunately did not have any material left after the PFAS analyses. For the conversion of concentrations in muscle, liver, kidney and spleen, we therefore used data on tissue/organ wet and dw from 64 bank vole specimens that were included in Ecke et al.^37^ (Supplementary Table S9). To achieve conversion factors between heart and dw in bank vole hearts, we used hearts from 19 bank voles trapped in spring and autumn 2022 near the city of Umeå, northern Sweden that we freeze-dried like the ungulate samples.

### Statistical analyses

For statistical analyses, concentrations that were below LOQ were replaced by half of LOQ. We only included PFAS in the statistical analyses that were detected in at least two samples. Based on the results of the analysed PFAS, we combined the respective PFAS to calculate the sum of all PFAS, PFAS4 (sum of PFOA, PFNA, PFHxS, and PFOS), PFCAs, PFSAs, and the sum of precursors.

To assess potential bioaccumulation of PFAS from soil to biota and biomagnification from mushrooms and berries to herbivores (bank voles and ungulates), we calculated bioaccumulation and biomagnification factors, for simplicity here referred to as biomagnification factors (BMFs) by dividing the concentration of the respective compound in biota at higher trophic level by its concentration in the environment or in biota at lower trophic level. We calculated BMFs from soil to biota, from mushrooms and berries to herbivores, and from voles to owls. BMFs > 1 were interpreted as bioaccumulation (from soil to biota) and biomagnification (from one biota to another).

To meet assumptions for parametric tests, concentrations were Box-Cox-transformed prior analyses. We used principal component analysis^77^ to reduce the PFAS that had no missing values to a few essential components. Only principal components (PCs) that explained more than 10% of the variance among the PFAS variables were considered.

We computed one-way ANOVA on PFAS concentrations on Frösön to analyse if concentrations (all expressed in dw) varied among matrices (soil, raspberry, blueberry, lingonberry, sweet tooth, bank vole muscle, kidney, heart, liver, and spleen, as well as roe deer and moose kidney, lung, and liver). Differences in PFAS concentrations between Frösön and the reference localities were analysed by one-sided *t*-tests. We used Mann-Whitney *U*-tests and Kruskal-Wallis tests on non-transformed values to test for differences among groups (sex, age, class of histopathological change). We calculated Spearman’s rank correlation coefficients for analysing relationships between PFAS concentrations in vole pools and individual histopathological change per pool (2–3 voles per pool) for all potential combinations of PFAS concentrations and cytoplasmatic change. We then analysed the percentage of the correlations that had a *p*-value < 0.05.

Statistical analyses were performed in R^78^ using the packages ‘car’, ‘stats’ and ‘vegan’ supported by visualisation with packages ‘ggplot2’ and ‘ggfortify’.

When relating identified PFAS concentrations to thresholds for food safety, we replaced all values below LOQ by 0.

## Supplementary Information

Below is the link to the electronic supplementary material.

Supplementary Material 1

Supplementary Material 2

Supplementary Material 3

Supplementary Material 4

Supplementary Material 5

Supplementary Material 6

Supplementary Material 7

Supplementary Material 8

Supplementary Material 9

Supplementary Material 10

## Data Availability

The data on the samples included in this study as well as the diagnostic results generated during this study are provided in the Supplementary Information.
