# Supplementary material for: Biomagnification and potential health effects of per- and polyfluoroalkyl substances (PFAS) in a terrestrial food web
Source: Sci Rep. 2025 Aug 23;15:31003. doi: 10.1038/s41598-025-16395-6 (PMC12375026; doi:10.1038/s41598-025-16395-6)
Supplement: Supplementary file 1 — Supplementary Material 1 [file 41598_2025_16395_MOESM1_ESM.pdf]

Supplementary Information

Biomagnification and potential health effects of per- and polyfluoroalkyl substances (PFAS) in a terrestrial food web

Frauke Ecke, Bjørnar Ytrehus, Magnus Evander, Birger Hörnfeldt, Alexandra Leijon, Jonas Malmsten, Aleksandra Skrobonja, Lutz Ahrens

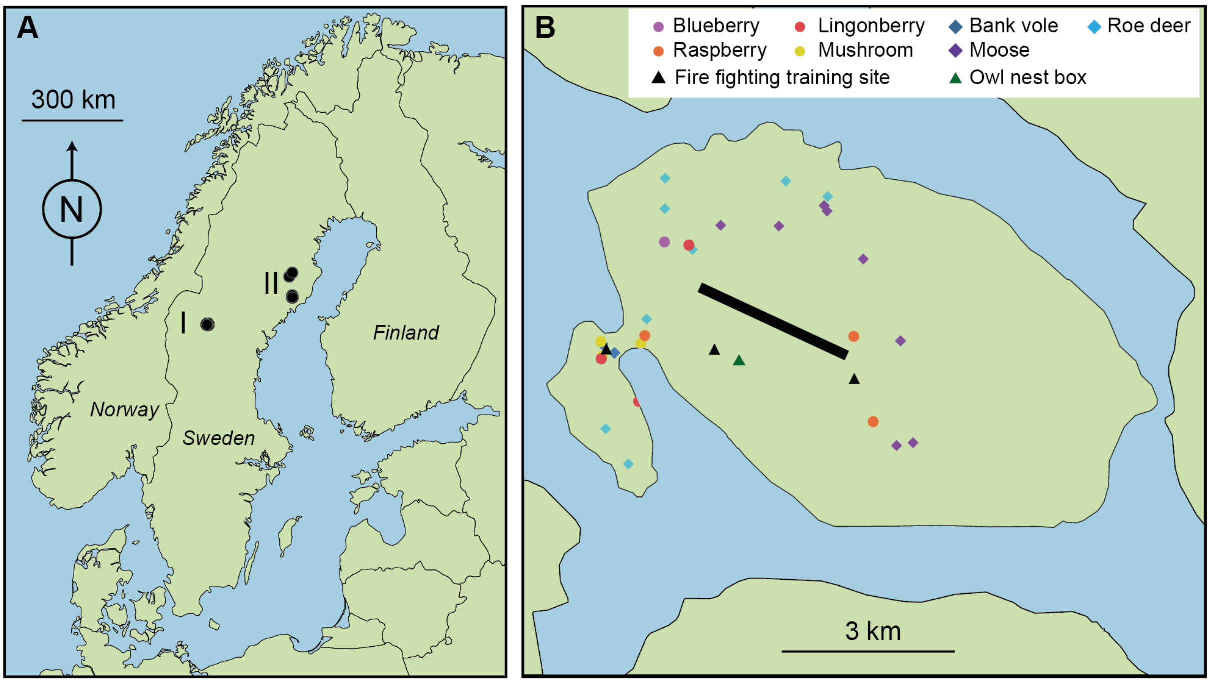

Supplementary Figure 1. **Sampling locations**. Map of Sweden and neighbouring countries showing (A) the study area on the island Frösön (I) and the reference area near the city of Umeå (II) and (B) a detailed map of the sampling localities (coloured filled circles) for the studied matrices on Frösön. The black line on Frösön in (B) represents the runway of Åre Östersund airport. Soil was sampled at the same sites where berries and mushrooms were sampled, except for some sites where only lingonberries were sampled.
