# Supplementary material for: Biomagnification and potential health effects of per- and polyfluoroalkyl substances (PFAS) in a terrestrial food web
Source: Sci Rep. 2025 Aug 23;15:31003. doi: 10.1038/s41598-025-16395-6 (PMC12375026; doi:10.1038/s41598-025-16395-6)
Supplement: Supplementary file 7 — Supplementary Material 7 [file 41598_2025_16395_MOESM7_ESM.pdf]

Biomagnification and potential health effects of per- and polyfluoroalkyl substances (PFAS) in a terrestrial food web

Frauke Ecke, Bjørnar Ytrehus, Magnus Evander, Birger Hörnfeldt, Alexandra Leijon, Jonas Malmsten, Aleksandra Skrobonja, Lutz Ahrens

Supplementary Table 8

Supplementary Table 8a. Individual PFAS limits of detection (LOD) and limits of quantification (LOQ) for biota samples and mushrooms, berries and soil.

PFAS	Wildlife		Berries		Soil		Mushrooms	
	LOD (ng/g ww)	LOQ (ng/g ww)	LOD (ng/g dw)	LOQ (ng/g dw)	LOD (ng/g dw)	LOQ (ng/g dw)	LOD (ng/g dw)	LOQ (ng/g dw)
PFBA	0.030	0.10	0.038	0.13	0.18	0.41	0.38	1.3
PFPeA	0.030	0.10	0.0076	0.03	0.15	0.26	0.85	0.98
PFHxA	0.04	0.13	0.038	0.13	0.24	0.43	0.04	0.13
PFHpA	0.030	0.10	0.038	0.13	0.14	0.34	0.38	1.3
PFOA	0.40	0.68	0.008	0.03	0.19	0.43	0.04	0.13
PFNA	0.30	1.0	0.038	0.13	0.038	0.13	0.38	1.3
PFDA	0.30	1.0	0.076	0.25	0.076	0.25	0.04	0.13
PFUnDA	0.08	0.3	0.076	0.25	0.19	0.22	0.41	0.76
PFDoDA	0.053	0.091	0.038	0.13	0.26	0.42	0.038	0.13
PFTriDA	0.060	0.20	0.038	0.13	0.093	0.14	0.038	0.13
PFTeDA	0.060	0.14	0.076	0.25	0.053	0.10	0.033	0.06
PFBS	0.15	0.31	0.069	0.12	0.98	1.6	0.51	1.1
PFPeS	0.060	0.20	0.008	0.025	0.076	0.25	0.076	0.25
L-PFHxS	0.030	0.10	0.076	0.25	0.25	0.46	0.30	0.69
B-PFHxS	0.012	0.04	0.015	0.05	0.07	0.23	0.30	0.69
PFHpS	0.04	0.1	0.038	0.13	0.008	0.025	0.076	0.25
L-PFOS	0.65	1.3	0.11	0.26	0.21	0.29	0.13	0.15
B-PFOS	0.12	0.40	0.015	0.051	0.036	0.036	0.13	0.15
PFNS	NA	NA	0.008	0.025	0.038	0.13	0.038	0.13
PFDS	0.060	0.20	0.038	0.13	0.038	0.13	0.038	0.13
FOSA	0.030	0.10	0.038	0.13	0.038	0.13	0.094	0.18
4:2 FTSA	NA	NA	0.038	0.13	0.24	0.28	0.38	1.3
6:2 FTSA	0.20	0.52	NA	NA	NA	NA	NA	NA
8:2 FTSA	0.30	1.0	0.076	0.25	0.91	2.1	0.08	0.11

NA = not analysed

Supplementary Table 8b. Recovery of individual PFAS in tissue samples (%).

PFAS	Recovery
PFBA	89
PFPeA	74
PFHxA	91
PFHpA	87
PFOA	93
PFNA	85
PFDA	81
PFUnDA	62
PFDoDA	80
PFTriDA	82
PFTeDA	84
PFBS	87
L-PFHxS	86
B-PFHxS	60
PFHpS	79
L-PFOS	100
B-PFOS	58
PFDS	86
FOSA	79
6:2 FTSA	89
8:2 FTSA	88

Supplementary Table 8c. Recovery of individual PFAS in soil samples (%).

PFAS	Recovery
PFBA	83
PFPeA	87
PFHxA	87
PFHpA	98
PFOA	108
PFNA	118
PFDA	84
PFUnDA	71
PFDoDA	116
PFTriDA	NA
PFTeDA	100
PFBS	102
L-PFHxS	98
PFPeS	91
B-PFHxS	55
PFHpS	93
L-PFOS	105
B-PFOS	44
PFNS	97
PFDS	87
FOSA	84
8:2 FTSA	95

NA = not available
