# Supplementary material for: Biomagnification and potential health effects of per- and polyfluoroalkyl substances (PFAS) in a terrestrial food web
Source: Sci Rep. 2025 Aug 23;15:31003. doi: 10.1038/s41598-025-16395-6 (PMC12375026; doi:10.1038/s41598-025-16395-6)
Supplement: Supplementary file 9 — Supplementary Material 9 [file 41598_2025_16395_MOESM9_ESM.pdf]

Biomagnification and potential health effects of per- and polyfluoroalkyl substances (PFAS) in a terrestrial food web

Frauke Ecke, Bjørnar Ytrehus, Magnus Evander, Birger Hörnfeldt, Alexandra Leijon, Jonas Malmsten, Aleksandra Skrobonja, Lutz Ahrens

Supplementary Table 3. Results of *t*-tests between PFAS concentrations on the island of Frösön and the reference localities near Umeå, northern Sweden. Missing values indicate that PFAS either were not analysed in the respective sample type or that concentrations were below limit of quantification (LOQ) at both sites.

PFAS	Sample type	Mean - Frösön	Mean - Reference	*t*-value	*df*	*P*
PFBA	Soil	0.19	0.56	-1.13	19	0.274
PFPeA	Soil	-0.39	-0.3	-0.15	19	0.886
PFHxA	Soil	-0.51	-0.85	0.79	19	0.438
PFHpA	Soil	-1.21	-1.32	0.4	19	0.697
PFOA	Soil	-0.76	-0.85	0.23	19	0.821
PFNA	Soil	-1.99	-1.67	-0.63	19	0.534
PFDA	Soil	-1.18	-1.06	-0.3	19	0.769
PFUnDA	Soil	-0.93	-0.23	-1.89	19	0.074
PFDoDA	Soil	-2.29	-1.79	-1.44	19	0.167
PFTriDA	Soil	-1.63	-1.08	-1.16	19	0.262
PFTeDA	Soil	-4.13	-2.82	-1.41	19	0.175
PFBS	Soil	0.01	-0.21	1.05	19	0.306
PFPeS	Soil	-1.91	-2.38	1.05	19	0.306
LPFHxS	Soil	-0.59	-1.52	1.84	19	0.081
BPFHxS	Soil	-2.85	-3.49	1.37	19	0.187
PFHpS	Soil	-4.43	-5.91	1.92	19	0.071
LPFOS	Soil	1.95	-0.46	3.63	19	0.002
BPFOS	Soil	-1.22	-3.1	2.72	19	0.014
PFNS	Soil	-2.14	-2.79	1.53	19	0.141
PFDS	Soil	-3.43	-4.42	1.57	19	0.132
FOSA	Soil	-4.69	-5.46	0.71	19	0.487
4:2-FTSA	Soil	-2.58	-2.58	0	19	0.996
6:2-FTSA	Soil				0	
8:2-FTSA	Soil	0.03	0.1	-0.95	19	0.353
∑PFCAs	Soil	1.45	1.66	-0.98	19	0.34
∑PFSAs	Soil	2.16	0.79	3.23	19	0.004
∑Precursors	Soil	0.41	0.35	0.52	19	0.609
∑PFAS	Soil	2.35	1.98	1.48	19	0.154
PFBA	Mushroom	4.11	4.10	0.018	4	0.987
PFPeA	Mushroom	0.95	0.47	1.598	4	0.185
PFHxA	Mushroom	-0.63	-0.75	0.072	4	0.946
PFHpA	Mushroom	-0.45	-0.45		4	
PFOA	Mushroom	-1.25	-2.35	1.000	4	0.374
PFNA	Mushroom	-0.48	-0.48		4	
PFDA	Mushroom	-2.37	-2.87	0.263	4	0.806

Appendix 3. Continued

PFAS	Sample type	Mean - Frösön	Mean - Reference	*t*-value	*df*	*P*
PFUnDA	Mushroom	-0.24	-1.12	1.000	4	0.374
PFDoDA	Mushroom	0.02	-4.57	2.503	4	0.067
PFTriDA	Mushroom	-2.90	-4.23	0.873	4	0.432
PFTeDA	Mushroom	-6.59	-7.34	0.158	4	0.882
PFBS	Mushroom	0.08	-0.65	1.000	4	0.374
PFPeS	Mushroom	-2.38	-2.38		4	
LPFHxS	Mushroom	-0.38	-1.16	1.000	4	0.374
BPFHxS	Mushroom	-2.87	-3.17	0.261	4	0.807
PFHpS	Mushroom	-2.39	-2.39		4	
LPFOS	Mushroom	-1.37	-0.95	-0.304	4	0.776
BPFOS	Mushroom	-3.71	-5.18	1.000	4	0.374
PFNS	Mushroom	-2.79	-2.79		4	
PFDS	Mushroom	-4.42	-4.42		4	
FOSA	Mushroom	-2.97	-5.22	1.975	4	0.119
4:2-FTSA	Mushroom	-0.51	-0.51		4	
6:2-FTSA	Mushroom				0	
8:2-FTSA	Mushroom	-1.15	-4.12	1.553	4	0.195
∑PFCAs	Mushroom	2.66	2.58	0.288	4	0.788
∑PFSAs	Mushroom	1.02	0.58	0.670	4	0.539
∑Precursors	Mushroom	0.52	-0.23	1.670	4	0.170
∑PFAS	Mushroom	2.80	2.69	0.382	4	0.722
PFBA	Raspberyy	0.71	-0.35	1.47	4	0.216
PFPeA	Raspberyy	-1.66	-2.53	0.91	4	0.413
PFHxA	Raspberyy	-0.97	-1.43	0.58	4	0.592
PFHpA	Raspberyy	-2.63	-2.63		4	
PFOA	Raspberyy	-2.07	-2.20	0.14	4	0.898
PFNA	Raspberyy	-3.70	-3.70		4	
PFDA	Raspberyy	-2.69	-2.69		4	
PFUnDA	Raspberyy	-2.88	-2.88		4	
PFDoDA	Raspberyy	-6.34	-6.34		4	
PFTriDA	Raspberyy	-7.78	-7.78		4	
PFTeDA	Raspberyy	-3.76	-3.76		4	
PFBS	Raspberyy	-3.25	-3.25		4	
PFPeS	Raspberyy	-5.94	-5.94		4	
LPFHxS	Raspberyy	-2.47	-2.47		4	
BPFHxS	Raspberyy	-8.50	-8.50		4	
PFHpS	Raspberyy	-3.36	-3.36		4	
LPFOS	Raspberyy	-2.12	-2.12		4	
BPFOS	Raspberyy	-4.33	-4.33		4	
PFNS	Raspberyy	-4.47	-4.47		4	
PFDS	Raspberyy	-4.42	-4.42		4	
FOSA	Raspberyy	-6.80	-6.80		4	
4:2-FTSA	Raspberyy	-6.25	-6.25		4	
6:2-FTSA	Raspberyy				0	
8:2-FTSA	Raspberyy	-2.86	-2.86		4	

Appendix 3. Continued

PFAS	Sample type	Mean - Frösön	Mean - Reference	*t*-value	*df*	*P*
∑PFCAs	Raspberyy	1.07	0.63	1.15	4	0.312
∑PFSAs	Raspberyy	-0.68	-0.68		4	
∑Precursors	Raspberyy	-1.85	-1.85		4	
∑PFAS	Raspberyy	1.26	0.94	1.16	4	0.311
PFBA	Blueberry	1.75	1.55	0.37	4	0.728
PFPeA	Blueberry	-0.89	-0.19	-0.57	4	0.599
PFHxA	Blueberry	-0.49	-0.60	0.25	4	0.812
PFHpA	Blueberry	-2.63	-2.63		4	
PFOA	Blueberry	-1.61	-1.01	-0.65	4	0.550
PFNA	Blueberry	-3.26	-3.70	1.00	4	0.374
PFDA	Blueberry	-2.69	-2.69		4	
PFUnDA	Blueberry	-2.88	-2.88		4	
PFDoDA	Blueberry	-6.34	-6.34		4	
PFTriDA	Blueberry	-7.78	-7.78		4	
PFTeDA	Blueberry	-3.76	-3.76		4	
PFBS	Blueberry	-2.50	-2.53	0.05	4	0.964
PFPeS	Blueberry	-5.94	-5.94		4	
LPFHxS	Blueberry	-2.47	-2.47		4	
BPFHxS	Blueberry	-8.50	-8.50		4	
PFHpS	Blueberry	-3.36	-3.36		4	
LPFOS	Blueberry	-2.12	-2.12		4	
BPFOS	Blueberry	-4.33	-4.33		4	
PFNS	Blueberry	-4.47	-4.47		4	
PFDS	Blueberry	-4.42	-4.42		4	
FOSA	Blueberry	-6.80	-6.80		4	
4:2-FTSA	Blueberry	-6.25	-6.25		4	
6:2-FTSA	Blueberry				0	
8:2-FTSA	Blueberry	-2.86	-2.86		4	
∑PFCAs	Blueberry	1.64	1.60	0.14	4	0.893
∑PFSAs	Blueberry	-0.57	-0.58	0.07	4	0.948
∑Precursors	Blueberry	-1.85	-1.85		4	
∑PFAS	Blueberry	1.74	1.71	0.14	4	0.892
PFBA	Lingonberry	-1.37	-0.80	-0.72	4	0.512
PFPeA	Lingonberry	-3.30	-3.30		4	
PFHxA	Lingonberry	-1.57	-1.03	-0.43	4	0.691
PFHpA	Lingonberry	-2.63	-2.63		4	
PFOA	Lingonberry	-1.73	-1.45	-2.21	4	0.092
PFNA	Lingonberry	-3.70	-3.70		4	
PFDA	Lingonberry	-2.69	-2.69		4	
PFUnDA	Lingonberry	-2.88	-2.88		4	
PFDoDA	Lingonberry	-6.34	-6.34		4	
PFTriDA	Lingonberry	-7.78	-7.78		4	
PFTeDA	Lingonberry	-3.76	-3.76		4	
PFBS	Lingonberry	-0.88	-0.88		4	
PFPeS	Lingonberry	-4.68	-5.94	1.00	4	0.374

Appendix 3. Continued

PFAS	Sample type	Mean - Frösön	Mean - Reference	*t*-value	*df*	*P*
LPFHxS	Lingonberry	-2.47	-2.47		4	
BPFHxS	Lingonberry	-8.50	-8.50		4	
PFHpS	Lingonberry	-3.36	-3.36		4	
LPFOS	Lingonberry	-2.12	-2.12		4	
BPFOS	Lingonberry	-4.33	-4.33		4	
PFNS	Lingonberry	-4.47	-4.47		4	
PFDS	Lingonberry	-4.42	-4.42		4	
FOSA	Lingonberry	-6.80	-6.80		4	
4:2-FTSA	Lingonberry	-6.25	-6.25		4	
6:2-FTSA	Lingonberry				0	
8:2-FTSA	Lingonberry	-2.86	-2.86		4	
∑PFCAs	Lingonberry	0.39	0.50	-0.25	4	0.815
∑PFSAs	Lingonberry	-0.07	-0.12	1.00	4	0.374
∑Precursors	Lingonberry	-1.85	-1.85		4	
∑PFAS	Lingonberry	0.95	0.96	0.00	4	0.998
