# Supplementary material for: Biomagnification and potential health effects of per- and polyfluoroalkyl substances (PFAS) in a terrestrial food web
Source: Sci Rep. 2025 Aug 23;15:31003. doi: 10.1038/s41598-025-16395-6 (PMC12375026; doi:10.1038/s41598-025-16395-6)
Supplement: Supplementary file 10 — Supplementary Material 10 [file 41598_2025_16395_MOESM10_ESM.pdf]

Biomagnification and potential health effects of per- and polyfluoroalkyl substances (PFAS) in a terrestrial food web

Frauke Ecke, Bjørnar Ytrehus, Magnus Evander, Birger Hörnfeldt, Alexandra Leijon, Jonas Malmsten, Aleksandra Skrobonja, Lutz Ahrens

Supplementary Table 2. Analysis of Variance (ANOVA) of different PFAS and their sums on Frösön showing sum of squares (SS), degrees of freedom (*df*), *F*-statistic (*F*) and *p*-values (*P*). Differences were tested among soil, fruits of lingonberry, blueberry, and raspberry, mushrooms, and tissues in bank voles (muscle, heart, lung, liver, kidney, and spleen), roedeer and moose (muscle, liver, kidney). The owl sample was not included in the statistical analysis due to *n* = 1.

PFAS	SS	*df*	*F*	*P*
PFBA	141.12	15	4.46	0.000
PFPeA	261.39	15	13.29	0.000
PFHxA	66.11	15	7.74	0.000
PFHpA	128.74	15	28.42	0.000
PFOA	147.53	15	15.78	0.000
PFNA	231.21	15	52.29	0.000
PFDA	183.30	15	38.41	0.000
PFUnDA	237.98	15	66.24	0.000
PFDoDA	326.84	15	41.58	0.000
PFTriDA	332.79	15	74.89	0.000
PFTeDA	227.18	15	21.19	0.000
PFBS	39.26	15	6.93	0.000
PFPeS	192.13	15	14.54	0.000
L-PFHxS	631.29	15	39.13	0.000
B-PFHxS	236.36	15	6.23	0.000
PFHpS	724.58	15	56.85	0.000
L-PFOS	1388.29	15	64.53	0.000
B-PFOS	1151.02	14	58.16	0.000
PFNS	900.73	15	133.91	0.000
PFDS	488.37	15	44.75	0.000
FOSA	0.61	4	14.56	0.000
4:2 FTSA	278.96	15	62.25	0.000
6:2 FTSA	32.88	10	15.65	0.000
8:2 FTSA	122.16	6	27.12	0.000
∑PFCAs	182.53	15	27.46	0.000
∑PFSAs	746.71	15	67.90	0.000
∑Precursors	184.29	15	74.15	0.000
∑PFAS	272.87	15	65.43	0.000
